# Efficient Noisy Sound-Event Mixture Classification Using Adaptive-Sparse Complex-Valued Matrix Factorization and OvsO SVM

**DOI:** 10.3390/s20164368

**Published:** 2020-08-05

**Authors:** Phetcharat Parathai, Naruephorn Tengtrairat, Wai Lok Woo, Mohammed A. M. Abdullah, Gholamreza Rafiee, Ossama Alshabrawy

**Affiliations:** 1 School of Software Engineering, Payap University, Chiang Mai 50000, Thailand; phetcharat@payap.ac.th (P.P.); naruephorn_t@payap.ac.th (N.T.); 2 Department of Computer and Information Sciences, Northumbria University, Newcastle upon Tyne NE1 8ST, UK; ossama.alshabrawy@northumbria.ac.uk; 3 Computer and Information Engineering Department, Ninevah University, Mosul 41002, Iraq; mohammed.abdulmuttaleb@uoninevah.edu.iq; 4 School of Electronics, Electrical Engineering and Computer Science, Queen’s University Belfast, Belfast BT9 5BN, UK; g.rafiee@qub.ac.uk

**Keywords:** audio signal processing, sound event classification, nonnegative matric factorization, blind signal separation, support vector machines

## Abstract

This paper proposes a solution for events classification from a sole noisy mixture that consist of two major steps: a sound-event separation and a sound-event classification. The traditional complex nonnegative matrix factorization (CMF) is extended by cooperation with the optimal adaptive *L*_1_ sparsity to decompose a noisy single-channel mixture. The proposed adaptive *L*_1_ sparsity CMF algorithm encodes the spectra pattern and estimates the phase of the original signals in time-frequency representation. Their features enhance the temporal decomposition process efficiently. The support vector machine (SVM) based one versus one (OvsO) strategy was applied with a mean supervector to categorize the demixed sound into the matching sound-event class. The first step of the multi-class MSVM method is to segment the separated signal into blocks by sliding demixed signals, then encoding the three features of each block. Mel frequency cepstral coefficients, short-time energy, and short-time zero-crossing rate are learned with multi sound-event classes by the SVM based OvsO method. The mean supervector is encoded from the obtained features. The proposed method has been evaluated with both separation and classification scenarios using real-world single recorded signals and compared with the state-of-the-art separation method. Experimental results confirmed that the proposed method outperformed the state-of-the-art methods.

## 1. Introduction

Surveillance systems have become increasingly ubiquitous in our living environment. These systems have been used in various applications including CCTV in traffic and site monitoring, and navigation. Automated surveillance is currently based on video sensory modality and machine intelligence. Recently, intelligent audio analysis has been taken into account in surveillance to improve the monitoring system via detection, classification, and recognition sound in a scenario. However, in a real-world situation, background noise has interfered in both the image and sound of a surveillance system. This will hinder the performance of a surveillance system. Hence, an automatic signal separation and event classification algorithm was proposed to improve the surveillance system by classifying the observed sound-event in noisy scenarios. The proposed noisy sound separation and event classification method is based on two approaches (i.e., blind signal separation and sound classification, which are introduced in the sections to follow, respectively).

The classical problem of blind source separation (BSS), the so-called “cocktail party problem”, is a psycho-acoustic spectacle that alludes to the significant human-auditory capability to selectively focus on and identify the sound-source speaker from the scenarios. The interference is produced by competing speech sounds or a variety of noises that are often assumed to be independent of each other. In the case of only a single microphone being available, this reduces to the single channel blind source separation (SCBSS) [1,2,3,4]. The majority of SCBSS algorithms work in time-frequency domain, for example, binary masking [5,6,7] or nonnegative matrix factorization (NMF) [8,9,10,11]. NMF has been continuously developed with great success for decomposing underlying original signals when a sole sensor is available. NMF was developed using the multiplicative update (MU) algorithm to solve its parametrical optimization based on a cost function such as the Kullback–Liebler divergence and the least square distance. Later, other families of cost functions have been continuously proposed, for example, the Beta divergence [12], Csiszar’s divergences, and Itakura–Saito divergence [13]. Additionally, iterative gradient update was presented where a sparsity constraint can be included into the optimizing function through regularization by minimizing penalized least squares [14] and using different sparsity constraints for dictionary and code [15]. The complex nonnegative matrix factorization (CMF) spreads the NMF model by combining a sparsity representation with the complex-spectrum domain to improve the audio separability. The CMF can extract the recurrent patterns of the phase estimates and magnitude spectra of constituent signals [16,17,18]. Nevertheless, the CMF lacks the generalized mechanics used for controlling the sparseness of the code. However, the sparsity parameter is manually determined for the above proposed methods. Approximate sparsity is an important consideration as they represent important information. Many sparse solutions have been proposed in the last decade [19,20,21,22,23,24,25]. Nonetheless, the optimal sparse solution remains an open issue.

Sound event classification (SEC) has vastly been exploited by many researchers. Sound can be categorized into speech, music, noise, environmental sound, or daily living sound [26]. Sound events are available in all classes, for example, car horn, traffic, walking, or knocking, etc. [27,28]. Sound-events contain significant information that can be used to describe what has happened or to predict what will happen next in the future. Most algorithms of the SEC methods are conveyed from sound classification approaches such as sparse coding, deep learning, and support vector machine (SVM). These approaches have been exploited to categorize a sound event in both indoor and outdoor scenarios. In recent years, the deep learning approach has been used to classify the sound-event. A deep learning framework can be established with two convolutional neural networks (CNNs) and a deep multi-layer perceptron (MLP) with rectified linear units (ReLU) as the activation function [29,30]. A Softmax function that is the final activation function is used to classify the sound into its corresponding class. The Softmax function is considered as the generalization of the logistic function, which aims to avoid overfitting. One of the advantages of deep learning is that it does not require feature extraction for the input sound. However, a deep neural network requires large training samples and despite a plethora of research, there is a general consensus that deep neural networks are still difficult to fine tune and generalize to test data. Moreover, it does not lend itself to the explanation as to why a certain decision is being made. Separate from the deep learning framework, another SEC approach is support vector machines [31,32], which has been practically presented to solve the classifier problem in various fields. The SVM algorithm relies on supervised learning by using the fundamental concept of statistic and risk minimization. The main process of the SVM is to draw the optimal separating hyperplane as the decision boundary located in such a way that the margin of separation between classes is maximized. The SVM approach is considered as supervised learning algorithm that is comprised of two sections: (1) a training section to model feature space and an optimal hyperplane, and (2) a testing section to use the SVM model for separating the observed data. The margin denotes the distance of the closest instance and the hyperplane. SVM has the desirable properties in that it requires only two differentiating factors to categorize two classes and a hyperplane that can be constructed to suit for an individual problem, even in the nonlinear case by selecting a kernel. Second, SVM provides a unique solution, since it is a convex optimization problem.

The rest of this paper is organized as follows. Section 2 presents the proposed noisy sound separation and event classification method, respectively. Next, Section 3 demonstrates and analyzes the performance of the proposed method. Finally, conclusions are drawn up in Section 4.

## 2. Background

**Figure 1:**
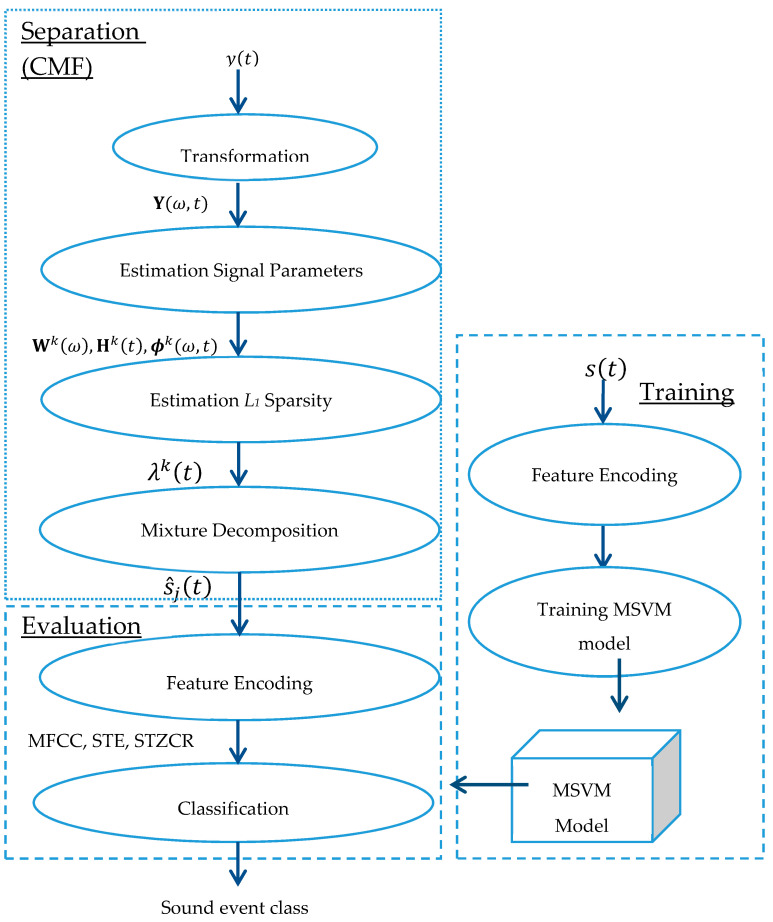
Signal flow of the proposed method.

Noisy mixed signals observed via a recording device can be stated as: $y\left(t\right)=\;s_{1}\left(t\right)+s_{2}\left(t\right)+n\left(t\right)$ where $s_{1}\left(t\right)$ and $s_{2}\left(t\right)$ denote the original sounds, and n(t) is noise. This research is focused on two sound events in a single recorded signal. The proposed method consists of two steps: noisy sound separation and sound event classification, which is illustrated in Figure 1, where y(t) and Y(ω,t) denote a sound-event mixture in the time domain and time-frequency domain, respectively. The terms $W^{k}\left(\omega \right),H^{k}\left(t\right),\;\phi^{k}\left(\omega ,t\right)$ are spectral basis, temporal code or weight matrix, and phase information, respectively. The term $\lambda^{k}\left(t\right)$ represents sparsity and ${\hat{s}}_{j}\left(t\right)$ is an estimated sound event source. The abbreviations MFCC, STE, and STZCR stand for Mel frequency cepstral coefficients, short-time energy, and short-time zero-crossing rate, respectively. The proposed method is consecutively elaborated in the following parts.

### 2.1. Single-Channel Sound Event Separation

The problem formulation in time-frequency (TF) representation is given by an observed complex spectrum, $Y_{f,t}\;\in \;\mathbb{C}$, to estimate the optimal parameters θ = {W,H, ϕ} of the model. A new factorization algorithm named as the adaptive *L*_1_-sparse complex non-negative matrix factorization (adaptive *L*_1_-SCMF) is derived in the following section. The generative model is given by

(1) $$Y\left(\omega ,t\right)=\overset{K}{\underset{k=1}{\sum }}W^{k}\left(\omega \right)H^{k}\left(t\right)Z^{k}\left(\omega ,t\right)=X\left(\omega ,t\right)+\epsilon \left(\omega ,t\right)$$

where $Z^{k}\left(\omega ,t\right)=e^{j\phi^{k}\left(\omega ,t\right)}$ and the reconstruction error $\epsilon \left(\omega ,t\right)\sim \;N_{\mathbb{C}}\left(0,\sigma^{2}\right)$ is assumed to be independently and identically distributed (i.i.d.) with white noise having zero mean and variance $\sigma^{2}$. The term ϵ(ω,t) is used to denote a modeling error for each source. The likelihood of θ = {W,H, ϕ} is thus written as

(2) $$P\left(Y|\theta \right)=\underset{f,t}{\prod }\frac{1}{\pi \sigma^{2}}\exp(-\frac{{\left|Y\left(\omega ,t\right)-\;X\left(\omega ,t\right)\right|}^{2}}{\sigma^{2}})$$

It is assumed that the prior distributions for W,H, and ϕ are independent, which yields

(3) P(θ|λ)= P(W)P(H|λ)P(ϕ) 

The prior P(H|λ) corresponds to the sparsity cost, for which a natural choice is a generalized Gaussian prior. When p=1, P(H|λ) promotes the $L_{1}$-norm sparsity. $L_{1}$-norm sparsity has been shown to be probabilistically equivalent to the pseudo-norm, $L_{0}$, which is the theoretically optimum sparsity [33,34]. However, $L_{0}$-norm is non-deterministic polynomial-time (NP) hard and is not useful in large datasets such as audio. Given Equation (3), the posterior density [35,36] is defined as the maximum a posteriori probability (MAP) estimation problem, which leads to minimizing the following optimization problem with respect to θ. Equations of Gaussian prior and maximum a posteriori probability (MAP) estimation are expressed in Appendix A.

The CMF parameters have been upgraded by using an efficient auxiliary function for an iterative process. The auxiliary function for f(θ) can be expressed as the following: for any auxiliary variables with $\underset{k}{\sum }{\bar{Y}}^{k}\left(\omega ,t\right)=Y\left(\omega ,t\right)$, for any $\beta^{k}\left(\omega ,t\right)>\;0$, $\underset{k}{\sum }\beta^{k}\left(\omega ,t\right)=1$, for any $H^{k}\left(t\right)\in ℛ,{\bar{H}}^{k}\left(t\right)\in ℛ$, and p=1. The term $f\left(\theta \right)\leq \;f^{+}\left(\theta ,\bar{\theta }\right)\;$ with an auxiliary function was defined as:

(4) $$f^{+}\left(\theta ,\bar{\theta }\right)\equiv \underset{f,k,t}{\sum }\frac{{\left|{\bar{Y}}^{k}\left(\omega ,t\right)-W^{k}\left(\omega \right)H^{k}\left(t\right)\cdot e^{j\phi^{k}\left(\omega ,t\right)}\right|}^{2}}{\beta^{k}\left(\omega ,t\right)}+\underset{k,t}{\sum }\left[{\left(\lambda^{k}\left(t\right)\right)}^{p}\left(p{\left|{\bar{H}}^{k}\left(t\right)\right|}^{p-2}H^{k}{\left(t\right)}^{2}+\left(2-p\right){\left|{\bar{H}}^{k}\left(t\right)\right|}^{p}\right)-\log\lambda^{k}\left(t\right)\right]$$

where $\bar{\theta }=\;\left\{{\bar{Y}}^{k}\left(\omega ,t\right),{\bar{H}}^{k}\left(t\right)|\;1\leq f\leq F,\;1\leq t\leq T,\;1\leq k\leq K\right\}$. The function $f^{+}\left(\theta ,\bar{\theta }\right)$ is minimized w.r.t. $\bar{\theta }$ when

(5) $${\bar{Y}}^{k}\left(\omega ,t\right)=\;W^{k}\left(\omega \right){\bar{H}}^{k}\left(t\right)\cdot e^{j\phi^{k}\left(\omega ,t\right)}+\;\beta^{k}\left(\omega ,t\right)\left(Y\left(\omega ,t\right)-X\left(\omega ,t\right)\right)$$

(6) $${\bar{H}}^{k}\left(t\right)=H^{k}\left(t\right)$$

### 2.2. Formulation of Proposed CMF Based Adaptive Variable Regularization Sparsity

#### 2.2.1. Estimation of the Spectral Basis and Temporal Code

In Equation (4), the update rule for θ is derived by differentiating $f^{+}\left(\theta ,\bar{\theta }\right)$ partially w.r.t. $W^{k}\left(\omega \right)$ and $H^{k}\left(t\right)$, and setting them to zero, which yields the following:

(7) $$W^{k}\left(\omega \right)\;=\frac{\sum_{t}\frac{H^{k}\left(t\right)}{\beta^{k}\left(\omega ,t\right)}Re\left[{\bar{Y}}^{k}{\left(\omega ,t\right)}^{*}\cdot e^{j\phi^{k}\left(\omega ,t\right)}\right]}{\sum_{t}\frac{H^{k}{\left(t\right)}^{2}}{\beta^{k}\left(\omega ,t\right)}}$$

(8) $$H^{k}\left(t\right)=\frac{\sum_{f}\frac{W^{k}\left(\omega \right)}{\beta^{k}\left(\omega ,t\right)}Re\left[{\bar{Y}}^{k}{\left(\omega ,t\right)}^{*}\cdot e^{j\phi^{k}\left(\omega ,t\right)}\right]}{\sum_{f}\frac{W^{k}{\left(\omega \right)}^{2}}{\beta^{k}\left(\omega ,t\right)}+\;{\left(\lambda^{k}\left(t\right)\right)}^{p}\;p{\left|{\bar{H}}^{k}\left(t\right)\right|}^{p-2}}$$

The update rule for the phase, $\phi^{k}\left(\omega ,t\right)$, can be derived by reformulating Equation (4) as follows:

(9) f+(θ,θ¯)=∑k,f,t|Y¯k(ω,t)|2− 2Wk(ω)Hk(t)Re[Y¯k(ω,t)·e−jϕk(ω,t)]+Wk(ω)2Hk(t)2βk(ω,t)+∑k,tλk(t)(|H¯k(t)|−1Hk(t)2−H¯k(t))−∑k,tlogλk(t) =A−2 ∑k,f,t|Bk(ω,t)|cos(ϕk(ω,t)−Ωk(ω,t))

where *A* denotes the terms that are irrelevant with $\phi^{k}\left(\omega ,t\right)$, $B^{k}\left(\omega ,t\right)=\frac{W^{k}\left(\omega \right)H^{k}\left(t\right){\bar{Y}}^{k}\left(\omega ,t\right)}{\beta^{k}\left(\omega ,t\right)}$, $\cos\Omega^{k}\left(\omega ,t\right)=\frac{Re\left[{\bar{Y}}^{k}\left(\omega ,t\right)\right]}{\left|{\bar{Y}}^{k}\left(\omega ,t\right)\right|}$, and sinΩk(ω,t)= Im[Y¯k(ω,t)]|Y¯k(ω,t)|. Derivation of (9) is elucidated in Appendix B. The auxiliary function, $f^{+}\left(\theta ,\bar{\theta }\right)$ in Equation (4) is minimized when $\cos\left(\phi^{k}\left(\omega ,t\right)-\Omega^{k}\left(\omega ,t\right)\right)=\cos\phi^{k}\left(\omega ,t\right)\cos\Omega^{k}\left(\omega ,t\right)+\;\sin\phi^{k}\left(\omega ,t\right)\sin\Omega^{k}\left(\omega ,t\right)=1$, namely, $\cos\phi^{k}\left(\omega ,t\right)=\cos\Omega^{k}\left(\omega ,t\right)\;$and $\sin\phi^{k}\left(\omega ,t\right)=\sin\Omega^{k}\left(\omega ,t\right)$. The update formula for $e^{j\phi^{k}\left(\omega ,t\right)}$ eventually leads to

(10) $$\begin{array}{cc} e^{j\phi^{k}\left(\omega ,t\right)} & =\cos\phi^{k}\left(\omega ,t\right)+j\sin\phi^{k}\left(\omega ,t\right)\\ & =\frac{{\bar{Y}}^{k}\left(\omega ,t\right)}{\left|{\bar{Y}}^{k}\left(\omega ,t\right)\right|} \end{array}$$

The update formula for $\beta^{k}\left(\omega ,t\right)\;$and $H^{k}\left(t\right)$ for projection onto the constraint space is set to

(11) $$\beta^{k}\left(\omega ,t\right)=\;\frac{W^{k}\left(\omega \right)H^{k}\left(t\right)}{\sum_{k}W^{k}\left(\omega \right)H^{k}\left(t\right)}\;$$

(12) $$H^{k}\left(t\right)\;\leftarrow \;\frac{H^{k}\left(t\right)}{\sum_{k}H^{k}\left(t\right)}$$

#### 2.2.2. Estimation of L_1_-Optimal Sparsity Parameter $\lambda^{k}\left(t\right)$

This section aims to facilitate spectral dictionaries with adaptive sparse coding. First, the CMF model is defined as the following terms:

(13) $$\begin{array}{c} \bar{W}=\left[I⊗W^{1}\left(\omega \right)\vdots I⊗W^{2}\left(\omega \right)\vdots \cdots \vdots I⊗W^{K}\left(\omega \right)\right],\\ e^{j\bar{\underline{\phi }}\left(t\right)}=\left[e^{j{\underline{\phi }}^{1}\left(t\right)}\vdots \cdots \vdots e^{j{\underline{\phi }}^{K}\left(t\right)}\right] \end{array}\;\underline{y}=\mathrm{vec}\left(Y\right)=\left[\begin{array}{c} {\underline{Y}}^{1}\left(:\right)\\ \ldots \\ {\underline{Y}}^{2}\left(:\right)\\ \ldots \\ \vdots \\ \ldots \\ {\underline{Y}}^{K}\left(:\right) \end{array}\right],\;\underline{h}=\left[\begin{array}{c} H^{1}\left(t\right)\\ \ldots \\ H^{2}\left(t\right)\\ \ldots \\ \vdots \\ \ldots \\ H^{K}\left(t\right) \end{array}\right],\;\underline{\lambda }=\left[\begin{array}{c} \lambda^{1}\left(t\right)\\ \ldots \\ \lambda^{2}\left(t\right)\\ \ldots \\ \vdots \\ \ldots \\ \lambda^{K}\left(t\right) \end{array}\right],\;\underline{\phi }=\left[\begin{array}{c} \phi^{1}\left(:,t\right)\\ \ldots \\ \phi^{2}\left(:,t\right)\\ \ldots \\ \vdots \\ \ldots \\ \phi^{K}\left(:,t\right) \end{array}\right]\;\bar{A}=\left[\begin{array}{cccc} \bar{W}{}^{\circ}e^{j\bar{\underline{\phi }}\left(t\right)} & 0 & \ldots & 0\\ 0 & \bar{W}{}^{\circ}e^{j\bar{\underline{\phi }}\left(t\right)} & 0 & \vdots \\ \vdots & 0 & \bar{W}{}^{\circ}e^{j\bar{\underline{\phi }}\left(t\right)} & 0\\ 0 & \ldots & 0 & \bar{W}{}^{\circ}e^{j\bar{\underline{\phi }}{\left(t\right)}_{t}} \end{array}\right]$$

where “⊗” and “°” are the Kronecker product and the Hadamard product, respectively. The term vec(∙) denotes the column vectorization and the term I is the identity matrix. The goal is then set to compute the regularization parameter $\lambda^{k}\left(t\right)$ related to each $H^{k}\left(t\right)$. To achieve the goal, the parameter p in Equation (4) is set to 1 to acquire a linear expression (in $\lambda^{k}\left(t\right)$). In consideration of the noise variance $\sigma^{2}$, Equation (4) can concisely be rewritten as:

(14) $$F\left(\underline{h},\underline{\lambda }\right)=\frac{1}{2\sigma^{2}}∥\underline{y}-\;\bar{A}\underline{h}{∥}_{F}^{2}+{\underline{\lambda }}^{T}\underline{h}-{\left(\log\underline{\lambda }\right)}^{T}\underline{1}$$

where the $\underline{h}$ and $\underline{\lambda }$ terms indicate vectors of dimension R×1 (i.e., R=F×T×K), and the superscript ‘T’ is used to denote complex Hermitian transpose (i.e., vector (or matrix) transpose followed by complex conjugate). The Expectation–Maximization (EM) algorithm will be used to determine $\underline{\lambda }$ and $\underline{h}$ is the hidden variable where the log-likelihood function can be optimized with respect to $\underline{\lambda }$. The log-likelihood function satisfies the following [12]:

(15) $$\ln p\left(\underline{y}\;|\underline{\lambda },\bar{A},\sigma^{2}\right)\;\geq \int Q\left(\underline{h}\right)\ln\left(\frac{p\left(\underline{y},\underline{h}|\underline{\lambda },\bar{A},\sigma^{2}\right)}{Q\left(\underline{h}\right)}\right)\;d\underline{h}$$

by applying the Jensen’s inequality for any distribution $Q\left(\underline{h}\right)$. The distribution can simply verify the posterior distribution of $\underline{h}$, which maximizes the right-hand side of Equation (15), is given by $Q\left(\underline{h}\right)=p\left(\underline{h}|\underline{y},\underline{\lambda },\bar{A},\sigma^{2}\right)$. The posterior distribution in the form of the Gibbs distribution is proposed as follows:

(16) $$Q\left(\underline{h}\right)=\;\frac{1}{Z_{h}}\exp\left[-F\left(\underline{h}\right)\right]\;\mathrm{where}\;Z_{h}=\int \exp\left[-F\left(\underline{h}\right)\right]d\underline{h}$$

The term $F\left(\underline{h}\right)$ in Equation (16) as the function of the Gibbs distribution is essential for simplifying the adaptive optimization of $\underline{\lambda }$. The maximum-likelihood (ML) estimation of $\underline{\lambda }$ can be decomposed as follows:

(17) $${\underline{\lambda }}^{ML}=\underset{\underline{\lambda }}{\arg\;\max}\int Q\left(\underline{h}\right)\ln p\left(\underline{h}|\underline{\lambda }\right)d\underline{h}$$

In the same way,

(18) $$\sigma^{2}{}_{ML}\;=\underset{\sigma^{2}}{\arg\;\max}\int Q\left(\underline{h}\right)\ln p\left(\underline{y}\;|\underline{h},\bar{A},\sigma^{2}\right)d\underline{h}$$

Individual element of H is required to be exponentially distributed with independent decay parameters that delivers $p\left(\underline{h}|\underline{\lambda }\right)=\;\underset{g}{\prod }\lambda_{g}\exp\left(-\lambda_{g}h_{g}\right)$, thus Equation (17) obtains

(19) $${\underline{\lambda }}^{ML}=\underset{\underline{\lambda }}{\arg\;\max}\int Q\left(\underline{h}\right)\left(\ln\lambda_{g}-\lambda_{g}h_{g}\;\right)d\underline{h}$$

The term $\underline{h}$ denotes the dependent variable of the distribution $Q\left(\underline{h}\right),$ whereas other parameters are assumed to be constant. As such, the $\underline{\lambda }$ optimization in Equation (19) is derived by differentiating the parameters within the integral with respect to $\underline{h}$. As a result, the functional optimization [37] of $\underline{\lambda }$ then obtains

(20) $$\lambda_{g}=\frac{1}{\int h_{g}Q\left(\underline{h}\right)d\underline{h}}$$

where g=1,2,…,R, $\lambda_{g}$ denotes the $g^{th}$ element of $\underline{\lambda }$. Notice that the solution $\underline{h}$ naturally splits its elements into distinct subsets ${\underline{h}}_{M}$ and ${\underline{h}}_{P}$, consisting of components $\forall_{m\;}\in M$ so that $h_{m}>0$ and components $\forall_{p\;}\in P$ so that $h_{P\;}=0$. The sparsity parameter is then obtained as presented in Equation (21):

(21) $$\lambda_{g\;}=\{\begin{array}{c} \frac{1}{\int h_{g}Q_{M}\left({\underline{h}}_{M}\right)d{\underline{h}}_{M}\;}=\frac{1}{h_{g}^{\mathrm{MAP}}}\;\mathrm{if}\;g\;\in \;M\\ \frac{1}{\int h_{g}{\hat{Q}}_{P}\left({\underline{h}}_{P}\right)d{\underline{h}}_{P}\;}=\frac{1}{u_{g}}\;\mathrm{if}\;g\;\in \;P \end{array}$$

and its covariance X is given by

(22) $$X_{ab\;}=\;\{\begin{array}{c} {\left({\bar{C}}_{P}^{-1}\right)}_{ab},\;\mathrm{if}\;a,b\;\in \;M\\ u_{p}^{2}\delta_{ab},\;\mathrm{Otherwise}. \end{array}$$

where ${\hat{Q}}_{P}\left({\underline{h}}_{P}\geq \;0\right)=\;\underset{p\in P}{\prod }\frac{1}{u_{p}}\exp\left(\frac{-h_{p}}{u_{p}}\right)$, ${\bar{C}}_{P}=\frac{1}{\sigma^{2}}{\bar{A}}_{P}^{T}{\bar{A}}_{P}$ and $u_{p}\leftarrow u_{p}\frac{-{\hat{b}}_{p}+\sqrt{{\hat{b}}_{p}^{2}+4\frac{{\left(\hat{C}\underline{u}\right)}_{p}}{{\tilde{u}}_{p}}}}{2{\left(\hat{C}\underline{u}\right)}_{p}}$. The function $Q_{M}\left({\underline{h}}_{M}\right)$ will be expressed as the unconstrained Gaussion with mean ${\underline{h}}_{M}^{\mathrm{MAP}}$ and covariance ${\bar{C}}_{M}^{-1}$ based on a multivariate Gaussian distribution. Similarly, the inference for $\sigma^{2}$ can be computed as

(23) $$\sigma^{2}=\frac{1}{N_{0}}\int Q\left(\underline{h}\right)\left(\|\underline{y}-\;\bar{A}\underline{h}{\|}^{2}\right)d\underline{h}$$

where

$${\hat{h}}_{g}=\{\begin{array}{c} \;h_{g}^{\mathrm{MAP}}\;\mathrm{if}\;g\;\in \;M\\ u_{g}\;\mathrm{if}\;g\;\in \;P \end{array}$$

The core procedure of the proposed CMF method is based on $L_{1}$-optimal sparsity parameters. The estimated sources are discovered by multiplying the respective rows of the $W^{k}\left(\omega \right)$ components with the corresponding columns of the $H^{k}\left(t\right)$ weights and time-varying phrase spectrum $e^{j\phi^{k}\left(\omega ,t\right)}$. The separated source ${\hat{s}}_{j}\left(t\right)$ is obtained by converting the time-frequency represented sources into the time domain. Derivation of *L*_1_-optimal sparsity parameter, is elucidated in the Appendix C.

### 2.3. Sound Event Classification

Once the separated sound signal is obtained, the next step is to identify the sound event. A multiclass support vector machine (MSVM) is employed to achieve the goal. The MSVM is comprised of two phases: the learning phase and the evaluation phase. The MSVM is based on one versus one strategy (OvsO) that splits observed c classes into $\frac{c\left(c-1\right)}{2}$ binary classification sub-problems. To train the ${𝓌}^{th}$ MSVM model, the MSVM will construct hyperplanes for discriminating each observed data into its corresponding class by executing the series of binary classification. Starting from the learning phase, sound signatures are extracted from the training dataset in the time-frequency domain. The sound signatures that were studied in this research were the Mel frequency cepstral coefficients (MFCC: MF), short-time energy (STE: E(t)), and short term zero-crossing rate (STZCR: STZ(t)), which can be orderly expressed as: MF=2525×log[1+(f/7)], $E\left(t\right)\;=\overset{\infty }{\underset{\tau =-\infty }{\sum }}{\left[y\left(t\right)\cdot f_{w}\left(t-\tau \right)\right]}^{2}$, $Z\left(t\right)\;=\overset{\infty }{\underset{\tau =-\infty }{\sum }}\left|sgn\left[s\left(\tau \right)\right]-sgn\left[s\left(\tau -1\right)\right]\right|\cdot f_{w}\left(t-\tau \right)$ where $f_{w}\left(t\right)$ denotes the windowing function. The training signals are segmented into small blocks, then the individual block is extracted to the three signature parameters. The mean supervector is then computed as an average of individual feature of all blocks for each sound event input. Thus, the mean feature supervector (O) with a corresponding sound-event-label vector ((w)) is paired together (i.e., (ψ(O,w))) and supplied to the MSVM model. The discriminant formula can be expressed as:

(24) $$\begin{array}{cc} \left\{\hat{𝓌},\beta \right\} & =\underset{𝓌}{\arg\;\max}\left\{\alpha_{𝓌}^{T}\psi \left(O,w;\beta \right)\right\}\\ & =\underset{𝓌}{\arg\;\max}\left\{\underset{\beta }{\max}\overset{\left|w\right|}{\underset{i=1}{\sum }}\alpha_{𝓌}^{T}\psi \left(O_{i|\beta },w_{i}\right)\right\} \end{array}$$

where $\left(O_{i|\beta },w_{i}\right),i=1,\ldots ,M$ represents the $i^{th}$ separated sound signals; the weight vector $\alpha_{𝓌}$ is employed for individual class 𝓌 to compute a discriminant score for the O; the i term is the index of the block order (β); and the function $\alpha_{𝓌}^{T}\psi \left(O,w;\beta \right)$ measures a linear discriminant distance of the hyperplane with the extracted feature vector from the observed data. The MSVM based OvsO strategy for class ${𝓌}^{th}$ and other, the hyperplane, can be maximized as $\alpha_{𝓌}^{T}\psi \left(O,w;\beta \right)+b_{𝓌}$ and can then be learned via the following equation as

(25) $$\underset{\alpha_{𝓌},\xi_{𝓌}}{\min}\frac{1}{2}\|\alpha_{𝓌}{\|}^{2}+C\overset{M}{\underset{i=1}{\sum }}\xi_{i}^{𝓌}$$

where $\xi_{i}^{𝓌}\geq 0$, $b_{𝓌}$ is a constant. The term $\overset{M}{\underset{i=1}{\sum }}\xi_{i}^{𝓌}$ denotes a penalty function for tradeoff between a large margin and a small error penalty. The optimal hyperplane can be determined by minimizing $\frac{1}{2}\|\alpha_{𝓌}{\|}^{2}$ to maximize the condition (i.e., $\alpha_{𝓌}^{T}\psi \left(O,w;\beta \right)+b_{𝓌}\;\geq 1-\xi_{i}^{𝓌})$. If the conditional term is greater than $1-\xi_{i}^{𝓌}$, then the estimated sound event belongs to the ${𝓌}^{th}$ class. Otherwise, the estimated sound event classifies into other classes.

The overview of the proposed algorithm is presented in the following table as Algorithm 1.

**Algorithm 1** Overview of the proposed algorithm.
(1)Compute Y(ω,t)=STFT(y(t)) from the noisy single-channel mixture y(t).(2)Initial values $W^{k}\left(\omega \right),H^{k}\left(t\right)$, $\;\beta^{k}\left(\omega ,t\right)$, fixing the value of $\phi^{k}\left(\omega ,t\right)$ at $e^{j\phi^{k}\left(\omega ,t\right)}\;=\frac{Y\left(\omega ,t\right)}{\left|Y\left(\omega ,t\right)\right|}$, and calculate $\lambda^{k}\left(t\right)$ and $\sigma^{2\;}$.(3)Update $\bar{\theta }=\;\left\{\;\bar{X},\bar{H}\right\}$, $\theta \;=\;\left\{W^{k}\left(\omega \right),H^{k}\left(t\right),\;\phi^{k}\left(\omega ,t\right)\right\}$, $\beta^{k}\left(\omega ,t\right)$.(4)Update parameters (21) and (23) until convergence is reached as determined by the rate of change of the parameters update falling within a pre-determined threshold.(5)Estimation of each source by multiplying the respective rows of the spectral components $W^{k}\left(\omega \right)$ with the corresponding columns of the mixture weights $H^{k}\left(t\right)$ and time-varying phrase spectrum $e^{j\phi^{k}\left(\omega ,t\right)}$. (i.e., ${\left|{\hat{S}}_{i}\right|}^{.2}=\overset{K_{i}}{\underset{k=1}{\sum }}W_{i}{}_{f}^{k}H_{i}{}_{t}^{k}\cdot e^{{j\phi }_{i}{}_{f,t}^{\left(k\right)}}$ and construct the binary TF mask for the $i^{th}$ source $M_{i}\left(f,t_{s}\right)∶=\{\begin{array}{c} \begin{array}{cc} 1, & \mathrm{if}\;{\left|{\hat{S}}_{i}\left(f,t_{s}\right)\right|}^{.2}>\;{\left|{\hat{S}}_{j}\left(f,t_{s}\right)\right|}^{.2},\;i\neq j \end{array}\\ \begin{array}{cc} 0, & otherwise\; \end{array} \end{array}\;$).(6)Convert the time-frequency represented sources into time domain to obtain the separated sources ${\hat{s}}_{j}\left(t\right)$ i.e., ${\hat{s}}_{j}\left(t\right)=\;STFT^{-1}\left({\left|{\hat{S}}_{i}\right|}^{2}\right)$.(7)Classify the ${𝓌}^{th}$ sound event by computing the optimal hyperplane $\alpha_{𝓌}^{T}\psi \left(O,w;\beta \right)+b_{𝓌}$ by minimizing the following equation: $\underset{\alpha_{𝓌},\xi_{𝓌}}{\min}\frac{1}{2}\|\alpha_{𝓌}{\|}^{2}+C\overset{M}{\underset{i=1}{\sum }}\xi_{i}^{𝓌}$.


## 3. Experimental Results and Analysis

The performance was evaluated on recorded sound-event signals in a low noisy environment at 20 signal-to-noise ratios (SNRs). The sound-event database had a total of 500 recorded signals containing four event classes: speech (SP), door open (DO), door knocking (DK), and footsteps (FS). An overview of the experimental setup is given as the following: all signals had a 16-bit resolution and a sampling frequency of 44.1 KHz. A 2048 length of Hanning window with 50% overlap was used for signal processing. Nonlinear SVM with a Gaussian RBF kernel was used for constructing the MSVM learning model. Other kernels such as polynomials, sigmoid, and even linear function were tested, but the best performance was delivered by the Gaussian kernel. A 4-fold cross-validation strategy was used in the training phase for tuning the classifier parameters when using 80% of the recorded signals (n = 400) from the sound-event database.

The performance of the proposed noisy sound separation and event classification (NSSEC) method was demonstrated and presented into the following two sections: (1) the separating performance, and (2) the MSVM classifier.

### 3.1. Sound-Event Separation and Classification Performance

Event mixtures consist of two sound-event signals in low noisy environment at 20 dB SNRs. A hundred sound-event signals of four classes were randomly selected and then mixed to generate 120 mixtures of six types (i.e., DO + DK, DO + FS, DO + SP, DK + FS, DK + SP, and FS + SP). The separation performance measured the signal-to-distortion ratio (SDR) (i.e., $SDR=10\;log_{10}\left(\|s_{target}{\|}^{2}/\|e_{interf}+e_{noise}+e_{artif}{\|}^{2}\right)$ where $e_{interf}$, $e_{noise}$, and $e_{artif}$). The SDR represents the ratio of the magnitude distortion of the original signal by the interference from other sources. The proposed separation method was compared with the state-of-the-art NMF approach (i.e., CMF [38], NMF-ISD [14,39], and SNMF [40,41,42] methods). The cost function was the least squares with 500 maximum number of iterations.

#### 3.1.1. Variational Sparsity Versus Fixed Sparsity

In this implementation, several experiments were conducted to investigate the effect of sparsity regularization on source separation performance. The proposed separation method was evaluated by variational sparsity in the case of (1) uniform constant sparsity with low sparseness e.g.,$\;\lambda_{t}^{k}=\;0.01$ and (2) uniform constant sparsity with high sparseness (e.g., $\lambda_{t}^{k}=\;100)$. The hypothesis is that the proposed variational sparsity will significantly yield improvement of the audio source separation when compared with fixed sparsity.

**Figure 2:**
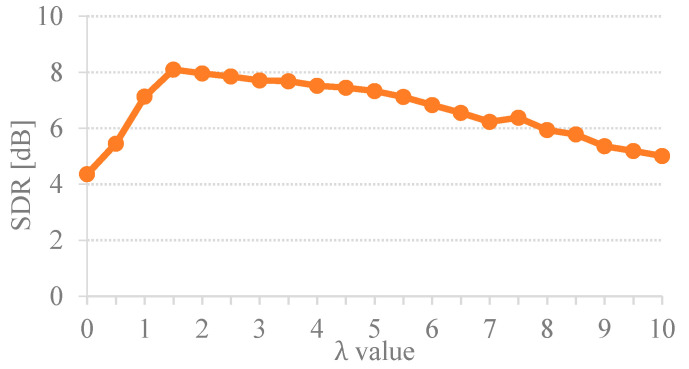
Separation results of the proposed method by using different uniform regularization.

To investigate the impact of uniform sparsity parameter, the set of sparsity regularization values from 0 to 10 with a 0.5 interval were determined for each experiment of 60 mixtures of six types. Results of the uniform regularization given by various sparsity (i.e., $\lambda_{t}^{k}=\;0,\;0.5,\;\ldots ,\;10$) is illustrated in Figure 2.

**Figure 3:**
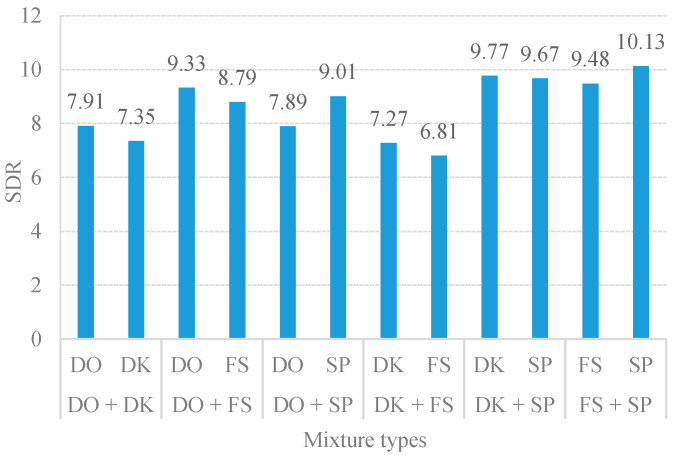
Average SDR results of six-mixture types.

Figure 2 illustrates that the best performance of the unsupervised CMF was in a range of 1.5–3, which yielded the highest SDR of over 8dB. When the term $\lambda_{t}^{k}$ was set too high, the low spectral values of sound-event signals were overly sparse. This overfitting sparsity $H^{k}\left(t\right)$ caused the separation performance toward a tendency to degrade. Conversely, the underfitting sparsity $H^{k}\left(t\right)$ occurred when the term $\lambda_{t}^{k}$ was set too low. The coding parameter $H^{k}\left(t\right)$ could not distinguish between the two sound-event signals. It was also noticed that if the factorization is non-regularized, this will cause the separation results to contain a mixed sound. According to the uniform sparsity results in Figure 2, the separation performance of the proposed method varies depending on the assigned sparsity values. Thus, it is challenging to find a solution for the indistinctness among the sound-event sources in the TF representation to determine the optimal value of sparseness. Thus, this introduces the importance of determining the optimal λ for separation. Table 1 presents the essential sparsity value on the separation performance by comparing the proposed method given by variational sparsity against the uniform sparsity scheme. The average performance improvement of the proposed adaptive CMF method against the uniform constant sparsity was 1.32 dB SDR. The SDR results clearly indicate that the adaptive sparsity yielded the surpass separation performance over the constant sparsity scheme. Hence, the proposed variational sparsity improves the performance of the discovered original sound-event signals by adaptively selecting the appropriate sparsity parameters to be individually adapted for each element code (i.e., $\lambda_{g\;}=\{\begin{array}{c} \frac{1}{\int h_{g}Q_{M}\left({\underline{h}}_{M}\right)d{\underline{h}}_{M}\;}=\frac{1}{h_{g}^{\mathrm{MAP}}}\;\mathrm{if}\;g\;\in \;M\\ \frac{1}{\int h_{g}{\hat{Q}}_{P}\left({\underline{h}}_{P}\right)d{\underline{h}}_{P}\;}=\frac{1}{u_{g}}\;\mathrm{if}\;g\;\in \;P \end{array}$ and $\sigma^{2}=\frac{1}{N_{0}}\int Q\left(\underline{h}\right)\left(\|\underline{y}-\;\bar{A}\underline{h}{\|}^{2}\right)d\underline{h}$ where ${\hat{h}}_{g}=\{\begin{array}{c} \;h_{g}^{\mathrm{MAP}}\;\mathrm{if}\;g\;\in \;M\\ u_{g}\;\mathrm{if}\;g\;\in \;P \end{array}$). Consequently, the optimal sparsity facilitates the estimated spectral dictionary via the estimated temporal code. The quantitative measures of separation performance were performed to assess the proposed single-channel sound event separation method. The overall average signal-to-distortion ratio (SDR) was 8.62 dB as illustrated in Figure 3.

**Figure 4:**
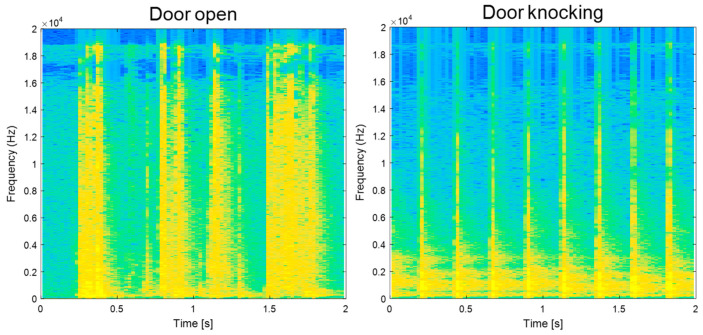

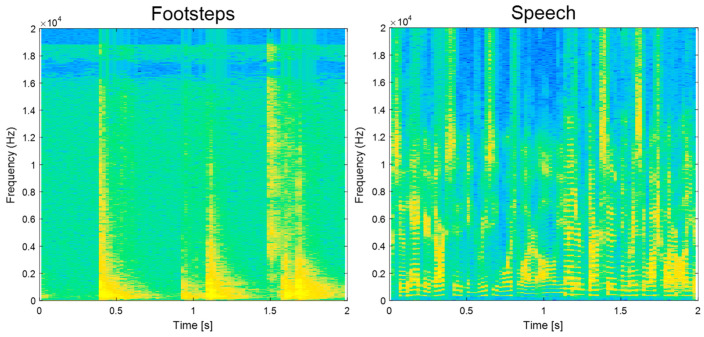
Example of time-frequency representation of four sound event classes.

Each sound-event signal has its own temporal pattern that can be clearly noticed in TF representation. Examples of sound-event signals in the TF domain are illustrated in Figure 4. Through the adaptive *L*_1_-SCMF method, the proposed single-channel separation method can generate complex temporal patterns such as speech. Thus, the separation results clearly indicate that the performances of noisy source separation perform with high SDR values.

#### 3.1.2. Comparison of the Proposed Adaptive CMF with Other SCBSS Methods Based on NMF

This section presents the adaptive CMF separating performance against the state-of-the-art NMF methods (i.e., CMF, SNMF, and NMF-ISD). In the compared methods, the experimental variables such as the normalizing time-frequency domain were computed by using the short-time Fourier transform (i.e., 1024-point Hanning window with 50% overlap). The number of factors was two, with a sparsity weight of 1.5. One hundred random realizations of twenty second-event mixtures were executed. As a result, the average SDRs are presented in Table 2. The proposed adaptive CMF method yielded the best separating performance over the CMF, SNMF, and NMF_ISD methods with the average improvement SDR at 2.13 dB. The estimated door open signals obtained the highest SDR among the four event categories.

The sparsity parameter was carefully adapted using the proposed adaptive *L_1_*-SCMF method exploiting the phase information and temporal code of the sources, which is inherently ignored by SNMF and NMF-ISD and has led to an improved performance of about 2 dB in SDR. On the other hand, the parts decomposed by the CMF, SNMF, and NMF-ISD methods were unable to capture the phase spectra and the temporal dependency of the frequency patterns within the audio signal.

Additionally, the CMF and NMF-ISD are unique when the signal adequately spans the positive octant. Thus, the rotation of W and opposite H can obtain the same results. The CMF method can easily be over or under sparse resolution of the factorization due to manually determining the sparsity value.

### 3.2. Performance of Event Classification Based on MSVM Algorithm

This section elucidates the features and performance of the MSVM-learning model. The MSVM-learning model was investigated to obtain the optimal size of the sliding window and then determine the significant features that led to the classification performance. Finally, the efficiency of the MSVM model was evaluated. These topics are presented in order in the following parts.

#### 3.2.1. Determination Optimal Window Length for Feature Encoding

For the MSVM method, sound-event signals are segmented into small blocks for encoding feature parameters by using a fixed-length of the sliding window. The sets of feature vectors are computed using the mean supervector and then loaded to the MSVM model for learning and constructing the hyperplane. The size of blocks can affect the information of the feature vectors, which leads to the classifier performance. The block’s size will affect the $\alpha_{𝓌}$, hence modifying the block size will mark the learning efficiency of the MSVM model. Therefore, in order to obtain the optimal value of $\alpha_{𝓌}$, the optimal block size was exploited by training the MSVM model given various lengths of window sizes (i.e., 0.5, 1, 1.5, and 2.0 s) to learn the 400 noisy sound-event signals of four event classes with cross-validation.

**Figure 5:**
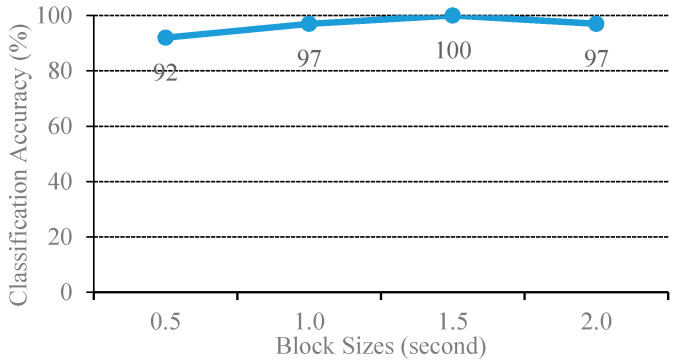
Classification performance of the original and combination source MSVM with various block sizes.

The experimental results are plotted in Figure 5, where the block size varies from 0.5 to 2.0 with 0.5 increments. The MSVM model of the 1.5 s block size yielded the best sound-event classification at 100% accuracy. The sliding window function benefits from SVM to learn an unknown sound event by generating the set of blocks from the observed event, regarded as a number of observed events. As a result, a set of sound event characteristics were computed for each block (i.e., $O_{i|\beta },w_{i}$ in Equation (24)).

The optimal length of the window size can capture the signature of the sound event. If the window length is too short, the encoded features will then deviate from the character of the sound event. In addition, the mean supervector is computed from the set of features of all blocks, which can be regarded as the mean of the probability distribution of the features. This mean supervector advantages the MSVM to reduce misclassifications when compared to the conventional SVM. Hence, the STFT of all experiments set the window function at 1.5 s.

#### 3.2.2. Determination of Sound-Event Features

**Figure 6:**
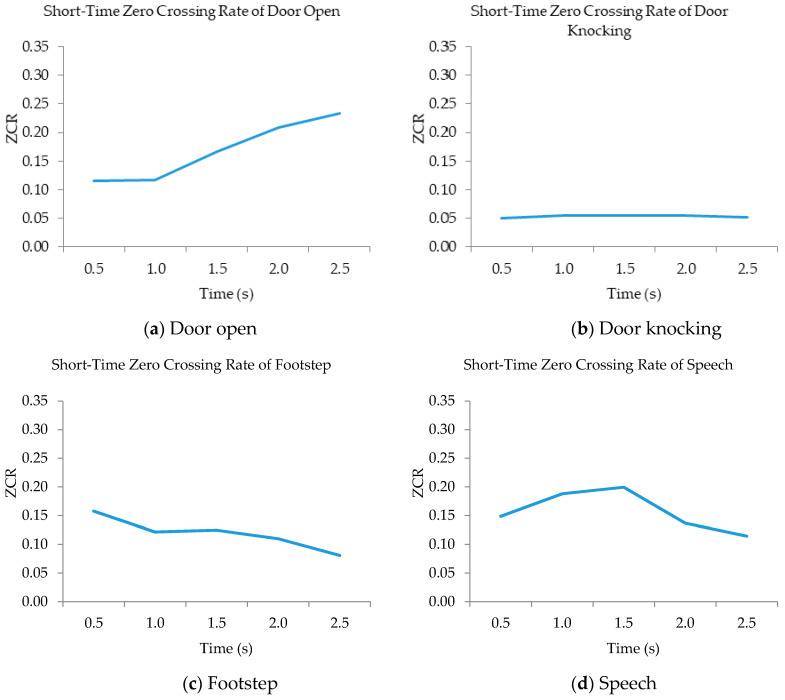
STZCR patterns of four sound-events (**a**–**d**).

Each sound-event signal was encoded with three features: Mel frequency cepstral coefficients (MFCCs), short-time energy (STE), and short-time zero-crossing rate (STZCR). MFCCs are represented as a frequency domain feature that is evaluated in a similar assembly to the human ear (i.e., logarithmic frequency perception). STE is the total spectrum power of an observed event. The STZCR denotes the number of times that the signal amplitude interval satisfies the condition (i.e., $STZCR=\;\left(1/T-1\right)\overset{T-1}{\underset{t=1}{\sum }}\left[[\{s_{t}s_{t-1}<0\right\}$ where $\left[[\{s_{t}s_{t-1}<0\right\}$ is 1 if the condition is true and 0 otherwise). The STZCR features of four sound-event classes are illustrated in Figure 6.

**Figure 7:**
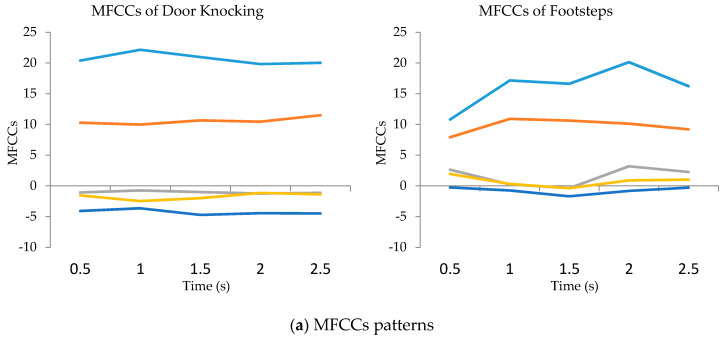

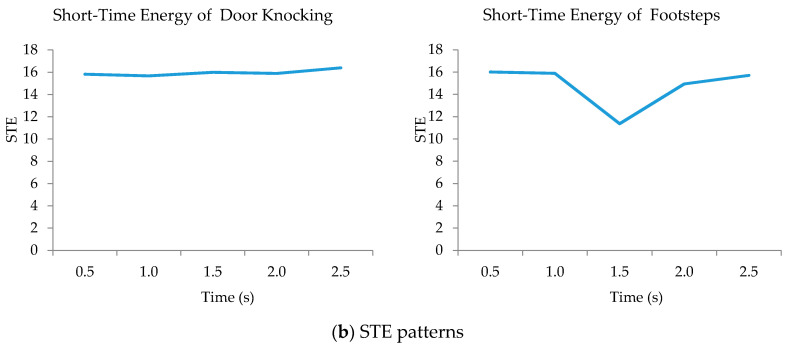
MFCCs (**a**) and STE (**b**) patterns of door knocking and footstep.

The STZCR feature represents unique patterns of four sound-event classes. The four sound-event patterns are different in shape and data range. Similarly, the MCFFs and STE features extract distinctive patterns of all event classes, except for the patterns between door knocking and footstep, as illustrated in Figure 7.

Figure 7 aims to compare the characteristics of similar sound events such as door knocking and footsteps. Thus, MFCCs and STE features were used to illustrate the patterns of sound events. Figure 7a represents the five orders of MFCC features to compare patterns between door knocking and walking while the STE features are shown in Figure 7b.

The proposed method separated the six categories of mixtures, then classified each estimated sound event signal into its corresponding class. Classified results of the six categories are presented as confusion matrixes below:

Actual






PredictDODK
DOFS
DOSPDO193DO128DO195DK315FS416SP313
DKFS
DKSP
FSSPDK124DK162FS146FS915SP517SP317

The classification of the proposed method was measured by Precision = TP/(TP + FP), Recall = TP/(TP + FN), and F1-score = 2 × (Precision × Recall)/(Precision + Recall). The TP and TN terms refer to the true positive and true negative, while the FP and FN terms mean false positive and false negative. The scores of Precision, Recall, and F1-score were 0.7667, 0.7731, and 0.7699, respectively.

Each feature represents unique characteristics of an individual sound event. Thus, features were matched into seven cases for exploiting their influence on the MSVM classifiers (i.e., {(MFCC), (STE), (STZCR), (MFCC, STE), (MFCC, STZCR), (STE, STZCR), (MFCC, STE, STZCR)}).

**Figure 8:**
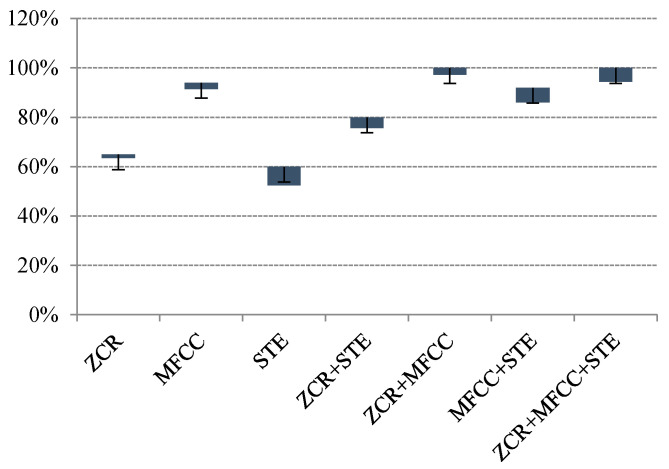
Classification performances of multi-class MSVM of various sets of features and length of event signal.

As shown in Figure 8, the MSVM model given by MFCCs and STZCR yielded the best classified accuracy at 100%, with less deviation among the other cases. Therefore, the separated signals were then classified by the proposed MSVM method given by the MFCC and STZCR vectors and the 1.5 s window function. The computational complexity of the proposed method was analyzed by two steps. First, the adaptive L1-SCMF method was NP-hard. Big-O of the adaptive *L*_1_-SCMF method consists of spectral basis (m), temporal code (n), and phase information that rely on components (k). Thus, Big-O of the separation step is ${\left(mn\right)}^{O\left(k^{2}\right)}$. For MSVM steps, it is a polynomial algorithm where Big-O is $O\left(n^{3}\right)$. Therefore, the computational complexity of the proposed method based on Big-O is ${\left(mn\right)}^{O\left(k^{2}\right)}$. All experiments were performed by a PC with Intel^®^ Core™ i7-4510U CPU2.00 GHz and 8 GB RAM. MATLAB was used as the programming platform.

#### 3.2.3. Performance of MSVM Classifier

The MSVM-classifier performance is presented in terms of percentage of the corrected sound-event classification. The 240 separated signals of four classes from the proposed separation method were individually identified by the MSVM classifier.

**Figure 9:**
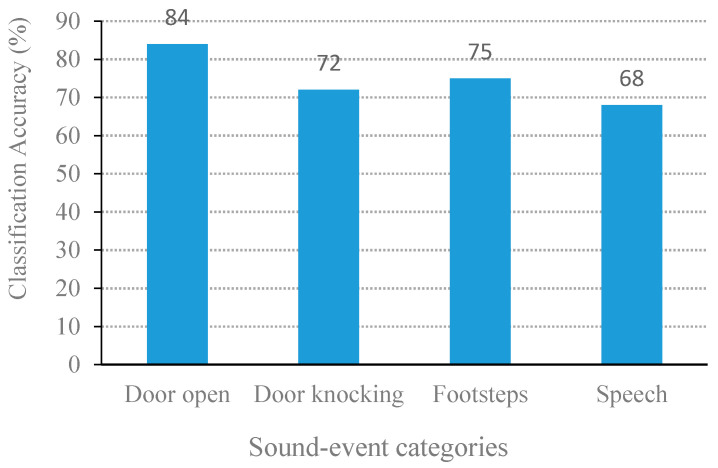
Average percentage of classification accuracy from the perspective of event group of the proposed NSSEC method.

**Figure 10:**
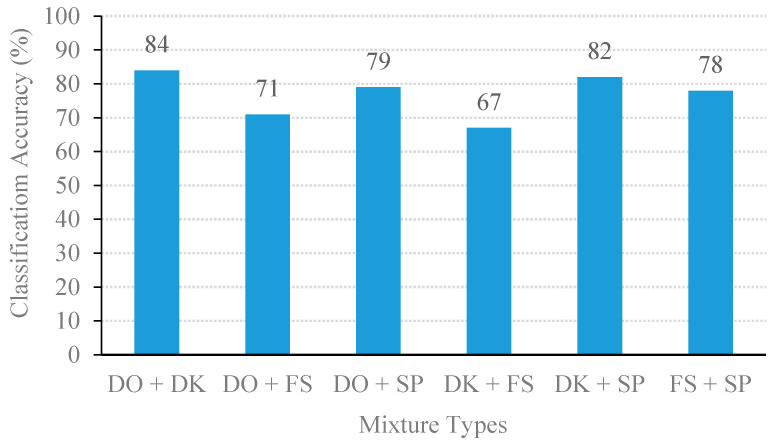
Classification performance of NSSEC model with 1.5 s block size.

Figure 9 compares the classification performance on the four classes of individual sound events. The best classification accuracy was door open, followed by footstep, door knocking, and speech. On the other hand, the classification results based on the mixed sound events are illustrated in Figure 10. The MSVM model delivered the highest performance of the door-open event with 84% accuracy.

From the above experiments, the proposed method yields an average classification accuracy of 76.67%. The MSVM method can well discriminate and classify the mixed event signals with high classification accuracy (i.e., the mixture of door open with door knocking and door knocking with speech were correctly classified with above 80% accuracy). Due to the MFCC and STZCR features in the individual event, these signals had obvious distinguishable patterns, as shown in the example of STZCR plots in Figure 6. Despite the SDR scores of the separated signals between door open and door knocking being relatively low (as given in Figure 3), the MSVM yielded the highest classification accuracy for the door open with door knocking mixture (DO + DK). This is attributed to the fact that interference remaining in the separated event signals causes the extracted MFCC and STZCR vectors to deviate from their original sound event vectors.

## 4. Conclusions

A novel solution for classification of the noisy mixtures using a single microphone was presented. The complex matrix factorization was proposed and extended by adaptively tuning the sparse regularization. Thus, the desired *L*_1_-optimal sparse decomposition was obtained. In addition, the phase estimates of the CMF could extract the recurrent pattern of the magnitude spectra. The updated equation was derived through an auxiliary function. For classification, the multiclass support vector was used as the mean supervector for encoding the sound-event signatures. The proposed noisy sound separation and event classification method was demonstrated by using four sets of noisy sound-event mixtures, which were door open, door knocking, footsteps, and speech. Based on the experimental results, first, the optimal window length of STFT was found where 1.5 s of the sliding window yielded the best separation performance. The second was two significant features that were ZCR and MFCCs. These parameters were set for examining the proposed method. The proposed method achieved outstanding results in both separation and classification. In future work, the proposed method will be evaluated on a public dataset such as the DCASE 2016, alongside the comparison with other machine learning algorithms.

## Figures and Tables

**Table 1:** Comparison of average SDR performance on three types of mixtures between uniform regularization methods and the proposed method.

Mixtures	Methods	SDR
DO + DK	Proposed method	7.63
	(Best) Uniform regularization sparsity	6.59
DO + FS	Proposed method	9.06
	(Best) Uniform regularization sparsity	8.74
DO + SP	Proposed method	8.45
	(Best) Uniform regularization sparsity	6.91
DK + FS	Proposed method	7.04
	(Best) Uniform regularization sparsity	6.35
DK + SP	Proposed method	9.72
	(Best) Uniform regularization sparsity	7.78
FS + SP	Proposed method	9.81
	(Best) Uniform regularization sparsity	7.42

**Table 2:** Comparison of average SDR and SIR performance on three types of mixtures between SCICA, NMF-ISD, SNMF, CMF, and the proposed method.

Mixtures	Methods	SDR
Door Open	Proposed method	8.38
	CMF	7.11
	SNMF	6.23
	NMF-ISD	6.17
Door Knocking	Proposed method	8.13
	CMF	7.06
	SNMF	6.52
	NMF-ISD	6.55
Footsteps	Proposed method	8.36
	CMF	7.89
	SNMF	6.62
	NMF-ISD	6.06
Speech	Proposed method	9.60
	CMF	6.73
	SNMF	5.61
	NMF-ISD	5.32

## Appendix A. Single-Channel Sound Event Separation

The prior P(H|λ) corresponds to the sparsity cost, for which a natural choice is a generalized Gaussian prior:

(A1) $$P\left(H|\lambda \right)=\;\underset{k,t}{\prod }\frac{p\lambda^{k}\left(t\right)}{2\Gamma \left(1/p\right)}\exp\;(-{\left(\lambda^{k}\left(t\right)\right)}^{p}{\left|H^{k}\left(t\right)\right|}^{p})$$

where $\lambda^{k}\left(t\right)$ and p are the shape parameters of the distribution. When p=1, P(H|λ) promotes the $L_{1}$-norm sparsity. $L_{1}$-norm sparsity has been shown to be probabilistically equivalent to the pseudo-norm, $L_{0}$, which is the theoretically optimum sparsity [29,30]. However, $L_{0}$-norm is non-deterministic polynomial-time (NP) hard and is not useful in large datasets such as audio. Given Equation (3), the posterior density is defined as

(A2) P(θ|Y,λ)∝P(Y|θ)P(H|λ) 

The maximum a posteriori probability (MAP) estimation problem leads to minimizing the following optimization problem with respect to θ:

(A3) $$f\left(\theta \right)=\;\underset{f,t}{\sum }{\left|Y\left(\omega ,t\right)-\;X\left(\omega ,t\right)\right|}^{2}+\;\underset{k,t}{\sum }\left[{\left(\lambda^{k}\left(t\right)\right)}^{p}{\left|H^{k}\left(t\right)\right|}^{p}-\log\lambda^{k}\left(t\right)\right]$$

subject to $\underset{f}{\sum }W^{k}\left(\omega \right)=1\;\left(k=1,\ldots ,K\right)$.

The CMF parameters has been upgraded by using an efficient auxiliary function for an iterative process. The auxiliary function for f(θ) can be expressed as the following: for any auxiliary variables with $\underset{k}{\sum }{\bar{Y}}^{k}\left(\omega ,t\right)=Y\left(\omega ,t\right)$, for any $\beta^{k}\left(\omega ,t\right)>\;0$, $\underset{k}{\sum }\beta^{k}\left(\omega ,t\right)=1$, for any $H^{k}\left(t\right)\in ℛ,{\bar{H}}^{k}\left(t\right)\in ℛ$, and p=1. The term $f\left(\theta \right)\leq \;f^{+}\left(\theta ,\bar{\theta }\right)\;$ with an auxiliary function was defined as

(A4) $$f^{+}\left(\theta ,\bar{\theta }\right)\equiv \underset{f,k,t}{\sum }\frac{{\left|{\bar{Y}}^{k}\left(\omega ,t\right)-W^{k}\left(\omega \right)H^{k}\left(t\right)\cdot e^{j\phi^{k}\left(\omega ,t\right)}\right|}^{2}}{\beta^{k}\left(\omega ,t\right)}+\underset{k,t}{\sum }\left[{\left(\lambda^{k}\left(t\right)\right)}^{p}\left(p{\left|{\bar{H}}^{k}\left(t\right)\right|}^{p-2}H^{k}{\left(t\right)}^{2}+\left(2-p\right){\left|{\bar{H}}^{k}\left(t\right)\right|}^{p}\right)-\log\lambda^{k}\left(t\right)\right]$$

where $\bar{\theta }=\;\left\{{\bar{Y}}^{k}\left(\omega ,t\right),{\bar{H}}^{k}\left(t\right)|\;1\leq f\leq F,\;1\leq t\leq T,\;1\leq k\leq K\right\}$. The function $f^{+}\left(\theta ,\bar{\theta }\right)$ is minimized w.r.t. $\bar{\theta }$ when

(A5) $${\bar{Y}}^{k}\left(\omega ,t\right)=\;W^{k}\left(\omega \right){\bar{H}}^{k}\left(t\right)\cdot e^{j\phi^{k}\left(\omega ,t\right)}+\;\beta^{k}\left(\omega ,t\right)\left(Y\left(\omega ,t\right)-X\left(\omega ,t\right)\right)$$

(A6) $${\bar{H}}^{k}\left(t\right)=H^{k}\left(t\right)$$

## Appendix B. Estimation of the Spectral Basis and Temporal Code

In Equation (4), the update rule for θ is derived by differentiating $f^{+}\left(\theta ,\bar{\theta }\right)$. partially w.r.t. $W^{k}\left(\omega \right)$ and $H^{k}\left(t\right)$, and setting them to zero, which yields the following:

(A7) $$W^{k}\left(\omega \right)\;=\frac{\sum_{t}\frac{H^{k}\left(t\right)}{\beta^{k}\left(\omega ,t\right)}Re\left[{\bar{Y}}^{k}{\left(\omega ,t\right)}^{*}\cdot e^{j\phi^{k}\left(\omega ,t\right)}\right]}{\sum_{t}\frac{H^{k}{\left(t\right)}^{2}}{\beta^{k}\left(\omega ,t\right)}}$$

(A8) $$H^{k}\left(t\right)=\frac{\sum_{f}\frac{W^{k}\left(\omega \right)}{\beta^{k}\left(\omega ,t\right)}Re\left[{\bar{Y}}^{k}{\left(\omega ,t\right)}^{*}\cdot e^{j\phi^{k}\left(\omega ,t\right)}\right]}{\sum_{f}\frac{W^{k}{\left(\omega \right)}^{2}}{\beta^{k}\left(\omega ,t\right)}+\;{\left(\lambda^{k}\left(t\right)\right)}^{p}\;p{\left|{\bar{H}}^{k}\left(t\right)\right|}^{p-2}}$$

The update rule for the phase, $\phi^{k}\left(\omega ,t\right)$, can be derived by reformulating Equation (A1) as follows:

(A9) f+(θ,θ¯)=∑k,f,t|Y¯k(ω,t)|2− 2Wk(ω)Hk(t)Re[Y¯k(ω,t)·e−jϕk(ω,t)]+Wk(ω)2Hk(t)2βk(ω,t)+∑k,tλk(t)(|H¯k(t)|−1Hk(t)2−H¯k(t))−∑k,tlogλk(t)=A−2 ∑k,f,tWk(ω)Hk(t)|Y¯k(ω,t)|βk(ω,t)(Re[Y¯k(ω,t)·e−jϕk(ω,t)]|Y¯k(ω,t)|)=A−2 ∑k,f,t|Bk(ω,t)|Re[(Y¯k(ω,t)(r)+jY¯k(ω,t)(i))(cosϕk(ω,t)−jsinϕk(ω,t))]|Y¯k(ω,t)|=A−2∑k,f,t|Bk(ω,t)|cosϕk(ω,t)cosΩk(ω,t)+ sinϕk(ω,t)sinΩk(ω,t)=A−2 ∑k,f,t|Bk(ω,t)|cos(ϕk(ω,t)−Ωk(ω,t))

where *A* denotes the terms that are irrelevant with $\phi^{k}\left(\omega ,t\right)$, $B^{k}\left(\omega ,t\right)=\frac{W^{k}\left(\omega \right)H^{k}\left(t\right){\bar{Y}}^{k}\left(\omega ,t\right)}{\beta^{k}\left(\omega ,t\right)}$, ${\cos\Omega }^{k}\left(\omega ,t\right)=\frac{Re\left[{\bar{Y}}^{k}\left(\omega ,t\right)\right]}{\left|{\bar{Y}}^{k}\left(\omega ,t\right)\right|}$, and sinΩk(ω,t)= Im[Y¯k(ω,t)]|Y¯k(ω,t)|. The auxiliary function, $f^{+}\left(\theta ,\bar{\theta }\right)$ in (A4) is minimized when $\cos\left(\phi^{k}\left(\omega ,t\right)-\Omega^{k}\left(\omega ,t\right)\right)=\cos\phi^{k}\left(\omega ,t\right)\cos^{k}\left(\omega ,t\right)+\;\sin\phi^{k}\left(\omega ,t\right){\sin\Omega }^{k}\left(\omega ,t\right)=1$, namely, $\cos\phi^{k}\left(\omega ,t\right)={\cos\Omega }^{k}\left(\omega ,t\right)\;$ and $\sin\phi^{k}\left(\omega ,t\right)={\sin\Omega }^{k}\left(\omega ,t\right)$. The update formula for $e^{j\phi^{k}\left(\omega ,t\right)}$ eventually leads to

(A10) ejϕk(ω,t)=cosϕk(ω,t)+jsinϕk(ω,t) =Re[Y¯k(ω,t)]+Im[Y¯k(ω,t)]|Y¯k(ω,t)| =Y¯k(ω,t)|Y¯k(ω,t)|

The update formula for $\beta^{k}\left(\omega ,t\right)\;$and $H^{k}\left(t\right)$ for projection onto the constraint space is set to

(A11) $$\beta^{k}\left(\omega ,t\right)=\;\frac{W^{k}\left(\omega \right)H^{k}\left(t\right)}{\sum_{k}W^{k}\left(\omega \right)H^{k}\left(t\right)}$$

(A12) $$H^{k}\left(t\right)\;\leftarrow \;\frac{H^{k}\left(t\right)}{\sum_{k}H^{k}\left(t\right)}$$

## Appendix C. Estimation of *L*_1_-Optimal Sparsity Parameter $\lambda^{k}\left(t\right)$

This section aims to facilitate spectral dictionaries with adaptive sparse coding. First, the CMF model is defined as the following terms:

(A13) $$\begin{array}{c} \bar{W}=\left[I⊗W^{1}\left(\omega \right)\vdots I⊗W^{2}\left(\omega \right)\vdots \cdots \vdots I⊗W^{K}\left(\omega \right)\right],\\ e^{j\bar{\underline{\phi }}\left(t\right)}=\left[e^{j{\underline{\phi }}^{1}\left(t\right)}\vdots \cdots \vdots e^{j{\underline{\phi }}^{K}\left(t\right)}\right] \end{array}\;\underline{y}=\mathrm{vec}\left(Y\right)=\left[\begin{array}{c} {\underline{Y}}^{1}\left(:\right)\\ \ldots \\ {\underline{Y}}^{2}\left(:\right)\\ \ldots \\ \vdots \\ \ldots \\ {\underline{Y}}^{K}\left(:\right) \end{array}\right],\;\underline{h}=\left[\begin{array}{c} H^{1}\left(t\right)\\ \ldots \\ H^{2}\left(t\right)\\ \ldots \\ \vdots \\ \ldots \\ H^{K}\left(t\right) \end{array}\right],\;\underline{\lambda }=\left[\begin{array}{c} \lambda^{1}\left(t\right)\\ \ldots \\ \lambda^{2}\left(t\right)\\ \ldots \\ \vdots \\ \ldots \\ \lambda^{K}\left(t\right) \end{array}\right],\;\underline{\phi }=\left[\begin{array}{c} \phi^{1}\left(:,t\right)\\ \ldots \\ \phi^{2}\left(:,t\right)\\ \ldots \\ \vdots \\ \ldots \\ \phi^{K}\left(:,t\right) \end{array}\right]\;\bar{A}=\left[\begin{array}{cccc} \bar{W}{}^{\circ}e^{j\bar{\underline{\phi }}\left(t\right)} & 0 & \ldots & 0\\ 0 & \bar{W}{}^{\circ}e^{j\bar{\underline{\phi }}\left(t\right)} & 0 & \vdots \\ \vdots & 0 & \bar{W}{}^{\circ}e^{j\bar{\underline{\phi }}\left(t\right)} & 0\\ 0 & \ldots & 0 & \bar{W}{}^{\circ}e^{j\bar{\underline{\phi }}{\left(t\right)}_{t}} \end{array}\right]$$

where “⊗” and “°” are the Kronecker product and the Hadamard product, respectively. The term vec(∙) denotes the column vectorization and the term I is the identity matrix. The goal is then set to compute the regularization parameter $\lambda^{k}\left(t\right)$ related to each $H^{k}\left(t\right)$. To achieve the goal, the parameter p in Equation (A3) was set at 1 to acquire a linear expression (in $\lambda^{k}\left(t\right)$). In consideration of the noise variance $\sigma^{2}$, Equation (A3) can concisely be rewritten as:

(A14) $$F\left(\underline{h},\underline{\lambda }\right)=\frac{1}{2\sigma^{2}}\underline{y}-\;\bar{A}{\underline{h}}_{F}^{2}+{\underline{\lambda }}^{T}\underline{h}-{\left(\log\underline{\lambda }\right)}^{T}\underline{1}$$

where the $\underline{h}$ and $\underline{\lambda }$ terms indicate vectors of dimension R×1 (i.e., R=F×T×K), and the superscript ‘T’ is used to denote complex Hermitian transpose (i.e., vector (or matrix) transpose), followed by complex conjugate. The Expectation–Maximization (EM) algorithm is used to determine $\underline{\lambda }$ and $\underline{h}$ is the hidden variable, where the log-likelihood function can be optimized with respect to $\underline{\lambda }$. The log-likelihood function satisfies the following [12]:

(A15) $$\ln p\left(\underline{y}\;|\underline{\lambda },\bar{A},\sigma^{2}\right)\;\geq \int Q\left(\underline{h}\right)\ln\left(\frac{p\left(\underline{y},\underline{h}|\underline{\lambda },\bar{A},\sigma^{2}\right)}{Q\left(\underline{h}\right)}\right)\;d\underline{h}$$

by applying the Jensen’s inequality for any distribution $Q\left(\underline{h}\right)$. The distribution can simply verify the posterior distribution of $\underline{h}$ that maximizes the right-hand side of Equation (A19) is given by $Q\left(\underline{h}\right)=p\left(\underline{h}|\underline{y},\underline{\lambda },\bar{A},\sigma^{2}\right)$. The posterior distribution in the form of the Gibbs distribution is proposed as follows:

(A16) $$Q\left(\underline{h}\right)=\;\frac{1}{Z_{h}}\exp\left[-F\left(\underline{h}\right)\right]\;\mathrm{where}\;Z_{h}=\int \exp\left[-F\left(\underline{h}\right)\right]d\underline{h}$$

The term $F\left(\underline{h}\right)$ in Equation (A16) as the function of the Gibbs distribution is essential for simplifying the adaptive optimization of $\underline{\lambda }$. The maximum-likelihood (ML) estimation of $\underline{\lambda }$ can be decomposed as follows:

(A17) $$\begin{array}{cc} {\underline{\lambda }}^{ML} & =\underset{\underline{\lambda }}{\arg\;\max}\ln p\left(\underline{y}\;|\underline{\lambda },\bar{A},\sigma^{2}\right)\\ & =\underset{\underline{\lambda }}{\arg\;\max}\int Q\left(\underline{h}\right)\left(\ln p\left(\underline{y}\;|\underline{h},\bar{A},\sigma^{2}\right)+\ln p\left(\underline{h}|\underline{\lambda }\right)\right)d\underline{h}\;\\ & =\underset{\underline{\lambda }}{\arg\;\max}\int Q\left(\underline{h}\right)\ln p\left(\underline{h}|\underline{\lambda }\right)d\underline{h} \end{array}$$

In the same way,

(A18) $$\begin{array}{cc} \sigma^{2}{}_{ML} & =\underset{\sigma^{2}}{\arg\;\max}\ln p\left(\underline{y}\;|\underline{\lambda },\bar{A},\sigma^{2}\right)\\ & =\underset{\sigma^{2}}{\arg\;\max}\int Q\left(\underline{h}\right)\left(\ln p\left(\underline{y}\;|\underline{h},\bar{A},\sigma^{2}\right)+\ln p\left(\underline{h}|\underline{\lambda }\right)\right)d\underline{h}\\ & =\underset{\sigma^{2}}{\arg\;\max}\int Q\left(\underline{h}\right)\ln p\left(\underline{y}\;|\underline{h},\bar{A},\sigma^{2}\right)d\underline{h} \end{array}$$

Individual element of H is required to be exponentially distributed with independent decay parameters that delivers $p\left(\underline{h}|\underline{\lambda }\right)=\;\underset{g}{\prod }\lambda_{g}\exp\left(-\lambda_{g}h_{g}\right)$, thus Equation (20) obtains

(A19) $${\underline{\lambda }}^{ML}=\underset{\underline{\lambda }}{\arg\;\max}\int Q\left(\underline{h}\right)\left(\ln\lambda_{g}-\lambda_{g}h_{g}\;\right)d\underline{h}$$

The term $\underline{h}$ denotes the dependent variable of the distribution $Q\left(\underline{h}\right)$ whereas other parameters are assumed to be constant. As such, the $\underline{\lambda }$ optimization in (A19) is derived by differentiating the parameters within the integral with respect to $\underline{h}$. As a result, the functional optimization of $\underline{\lambda }$ then obtains

(A20) $$\lambda_{g}=\frac{1}{\int h_{g}Q\left(\underline{h}\right)d\underline{h}}$$

where g=1,2,…,R, $\lambda_{g}$ denotes the $g^{th}$ element of $\underline{\lambda }$. The iterative update for $\sigma_{ML}^{2}$ is given by

(A21) $$\begin{array}{cc} \sigma_{ML}^{2} & =\underset{\sigma^{2}}{\arg\;\max}\int Q\left(\underline{h}\right)\left(\frac{-N_{0}}{2}\ln\left(\pi \sigma^{2}\right)-\frac{1}{2\sigma^{2}}\|\underline{y}-\;\bar{A}\underline{h}{\|}^{2}\right)d\underline{h}\\ & =\frac{1}{N_{0}}\int Q\left(\underline{h}\right)\left(\|\underline{y}-\;\bar{A}\underline{h}{\|}^{2}\right)d\underline{h} \end{array}$$

where $p\left(\underline{y}\;|\underline{h},\bar{A},\sigma^{2}\right)=\;{\left(\pi \sigma^{2}\right)}^{-N_{0}/2}\exp\left(-\left(1/2\sigma^{2}\right)\|\underline{y}-\;\bar{A}\underline{h}{\|}^{2}\right)$ and $N_{0}=\;K\times T$. However, the integral forms in Equations (A20) and (A21) are complex to compute and analyzed analytically. Thus, an approximation to $Q\left(\underline{h}\right)$ is exploited. Notice that the solution $\underline{h}$ naturally splits its elements into distinct subsets ${\underline{h}}_{M}$ and ${\underline{h}}_{P}$ consisting of components $\forall_{m\;}\in M$ such that $h_{m}>0$ and components $\forall_{p\;}\in P$ such that $h_{P\;}=0$. Hence, this can be derived as follows:

(A22) $$F\left(\underline{h},\underline{\lambda }\right)=F\left({\underline{h}}_{M},{\underline{\lambda }}_{M}\right)+F\left({\underline{h}}_{P},{\underline{\lambda }}_{P}\right)+G$$

Defined$\;F\left({\underline{h}}_{M},{\underline{\lambda }}_{M}\right)=\frac{1}{2\sigma^{2}}\|\underline{y}-\;{\bar{A}}_{M}{\underline{h}}_{M}{\|}^{2}+{\underline{\lambda }}_{M}^{T}{\underline{h}}_{M}-{\left(\log\underline{\lambda }\right)}_{M}^{T}\underline{1}M$, $F\left({\underline{h}}_{P},{\underline{\lambda }}_{P}\right)=\frac{1}{2\sigma^{2}}\|\underline{y}-\;{\bar{A}}_{P}{\underline{h}}_{P}{\|}^{2}+{\underline{\lambda }}_{P}^{T}{\underline{h}}_{P}-{\left(\log\underline{\lambda }\right)}_{P}^{T}\underline{1}P$, and $G=\frac{1}{2\sigma^{2}}\left[2{\left({\bar{A}}_{M}{\underline{h}}_{M}\right)}^{T}\left({\bar{A}}_{P}{\underline{h}}_{P}\right)-\|\underline{y}{\|}^{2}\right]$. Here, the term $\|\underline{y}{\|}^{2}$ is a constant and the cross-term ${\left({\bar{A}}_{M}{\underline{h}}_{M}\right)}^{T}\left({\bar{A}}_{P}{\underline{h}}_{P}\right)$ measures the orthogonality between ${\bar{A}}_{M}{\underline{h}}_{M}$ and ${\bar{A}}_{P}{\underline{h}}_{P}$, where ${\bar{A}}_{M}$ and ${\bar{A}}_{P}$ denote the sub-matrix of $\bar{A}$ that corresponds to ${\underline{h}}_{M}$ and ${\underline{h}}_{P}$. To obtain a simplified expression in Equation (A22), the $F\left(\underline{h}\right)$ function can be approximated as $F\left(\underline{h},\underline{\lambda }\right)\approx F\left({\underline{h}}_{M},{\underline{\lambda }}_{M}\right)+F\left({\underline{h}}_{P},{\underline{\lambda }}_{P}\right)$ and the G can be safely discounted since its value is typically much smaller than $F\left({\underline{h}}_{M},{\underline{\lambda }}_{M}\right)$ and $F\left({\underline{h}}_{P},{\underline{\lambda }}_{P}\right)$. Thus, the approximation of $Q\left(\underline{h}\right)$ can be expressed as

(A23) $$\begin{array}{cc} Q\left(\underline{h},\underline{\lambda }\right) & =\;\frac{1}{Z_{h}}\exp\left[-F\left(\underline{h},\underline{\lambda }\right)\right]\\ & \approx \frac{1}{Z_{h}}\exp\left[-\left(F\left({\underline{h}}_{M},{\underline{\lambda }}_{M}\right)+F\left({\underline{h}}_{P},{\underline{\lambda }}_{P}\right)\right)\right]\\ & =\frac{1}{Z_{M}}\exp\left[-F\left({\underline{h}}_{M},{\underline{\lambda }}_{M}\right)\right] \end{array}\;\begin{array}{c} \frac{1}{Z_{P}}\exp\left[-F\left({\underline{h}}_{P},{\underline{\lambda }}_{P}\right)\right]\\ \;=\;Q_{M}\left({\underline{h}}_{M}\right)Q_{P}\left({\underline{h}}_{P}\right) \end{array}$$

Defining $Z_{M}=\int \exp\left[-F\left({\underline{h}}_{M},{\underline{\lambda }}_{M}\right)\right]d{\underline{h}}_{M}$ and $Z_{P}\;=\;\int \exp\left[-F\left({\underline{h}}_{P},{\underline{\lambda }}_{P}\right)\right]\;d{\underline{h}}_{P}$. With the purpose of characterizing $Q_{P}\left({\underline{h}}_{P}\right)$, some positive deviation to ${\underline{h}}_{P}$ is needed to be allowed for, whereas the ${\underline{h}}_{P}$ values will reject all negative values due to CMF only accepting zero and positive values. Thus, ${\underline{h}}_{P}$ admits zero and positive values in $Q_{P}\left({\underline{h}}_{P}\right)$. The approximation of the distribution $Q_{P}\left({\underline{h}}_{P}\right)$ is then utilized in the Taylor expansion as the *maximum a posterior probability* (MAP) estimate. Therefore, with ${\underline{h}}^{\mathrm{MAP}}$, one obtains

(A24) $$\begin{array}{cc} Q_{P}\left({\underline{h}}_{P}\geq \;0\right) & \propto \;\exp\left\{-{\left[{\left(\frac{\partial \;F}{\partial \underline{h}}\right)|}_{{\underline{h}}^{\mathrm{MAP}}}\right]}_{P}^{T}{\underline{h}}_{P}-\frac{1}{2}{\underline{h}}_{P}^{T}{\bar{C}}_{P}{\underline{h}}_{P}\right\}\\ & =\;\exp\left[-{\left(\bar{C}{\underline{h}}^{\mathrm{MAP}}-\frac{1}{\sigma^{2}}{\bar{A}}^{T}\underline{y}+\;\underline{\lambda }\right)}_{P}^{T}{\underline{h}}_{P}-\frac{1}{2}{\underline{h}}_{P}^{T}{\bar{C}}_{P}{\underline{h}}_{P}\right] \end{array}$$

where ${\bar{C}}_{P}=\frac{1}{\sigma^{2}}{\bar{A}}_{P}^{T}{\bar{A}}_{P}$ and $\bar{C}=\frac{1}{\sigma^{2}}{\bar{A}}^{T}\bar{A}$. The integration of the term $Q_{P}\left({\underline{h}}_{P}\right)$ in Equation (A24) is hard to derive in its closed form expression for analytical evaluation, which subsequently prohibits inference of the sparsity parameters. A fixed form distribution is employed for computing variational approximate $Q_{P}\left({\underline{h}}_{P}\right)$. As a result, the closed form expression is obtained. Subsequently, the term ${\underline{h}}_{P}$ only takes on nonnegative values, so a suitable fixed form distribution is to use the factorized exponential distribution given by

(A25) $${\hat{Q}}_{P}\left({\underline{h}}_{P}\geq \;0\right)=\;\underset{p\in P}{\prod }\frac{1}{u_{p}}\exp\left(\frac{-h_{p}}{u_{p}}\right)$$

By minimizing the Kullback–Leibler divergence between $Q_{P}$ and ${\hat{Q}}_{P}$, the variational parameters $\underline{u}=\;\left\{u_{p}\right\}$ where ∀p ∈ P can be derived as:

(A26) $$\begin{array}{cc} \underline{u} & =\;\arg\;\underset{\underset{=}{u}}{\min}{\hat{Q}}_{P}\left({\underline{h}}_{P}\right)\ln\frac{{\hat{Q}}_{P}\left({\underline{h}}_{P}\right)}{Q_{P}\left({\underline{h}}_{P}\right)}d{\underline{h}}_{P}\\ & =\;\arg\;\underset{\underset{=}{u}}{\min}\;{\hat{Q}}_{P}\left({\underline{h}}_{P}\right)\left[\ln{\hat{Q}}_{P}\left({\underline{h}}_{P}\right)-\ln Q_{P}\left({\underline{h}}_{P}\right)\right]d{\underline{h}}_{P} \end{array}$$

Solving Equation (A26) for $u_{p}$ leads to the following update [37]:

(A27) $$u_{p}\leftarrow u_{p}\frac{-{\hat{b}}_{p}+\sqrt{{\hat{b}}_{p}^{2}+4\frac{{\left(\hat{C}\underline{u}\right)}_{p}}{{\tilde{u}}_{p}}}}{2{\left(\hat{C}\underline{u}\right)}_{p}}$$

The approximate distribution for components ${\underline{h}}_{M}$ can be obtained by substituting $F\left({\underline{h}}_{M},{\underline{\lambda }}_{M}\right)$ into $Q_{M}\left({\underline{h}}_{M}\right)$ as follows:

(A28) $$\begin{array}{cc} Q_{M}\left({\underline{h}}_{M}\right) & =\frac{1}{Z_{M}}\exp\left[-F\left({\underline{h}}_{M},{\underline{\lambda }}_{M}\right)\right]\\ & \propto \;\exp\left[-\left(\frac{1}{2}{\underline{h}}_{M}^{T}{\bar{C}}_{M}{\underline{h}}_{M}-\frac{1}{\sigma^{2}}{\underline{y}}^{T}{\bar{A}}_{M}{\underline{h}}_{M}+{\underline{\lambda }}_{M}{\underline{h}}_{M}\right)\right] \end{array}$$

In Equation (A28), the function $Q_{M}\left({\underline{h}}_{M}\right)$ will be expressed as the unconstrained Gaussion with mean ${\underline{h}}_{M}^{\mathrm{MAP}}$ and covariance ${\bar{C}}_{M}^{-1}$ based on a multivariate Gaussian distribution. The term ${\bar{C}}_{M}$ denotes the sub-matrix of $\bar{C}$. The sparsity parameter is then obtained by substituting Equations (A24), (A25), and (A28) into Equation (A20) as presented in Equation (A29):

(A29) $$\lambda_{g\;}=\{\begin{array}{c} \frac{1}{\int h_{g}Q_{M}\left({\underline{h}}_{M}\right)d{\underline{h}}_{M}\;}=\frac{1}{h_{g}^{\mathrm{MAP}}}\;\mathrm{if}\;g\;\in \;M\\ \frac{1}{\int h_{g}{\hat{Q}}_{P}\left({\underline{h}}_{P}\right)d{\underline{h}}_{P}\;}=\frac{1}{u_{g}}\;\mathrm{if}\;g\;\in \;P \end{array}$$

and its covariance X is given by

(A30) $$X_{ab\;}=\;\{\begin{array}{c} {\left({\bar{C}}_{P}^{-1}\right)}_{ab},\;\mathrm{if}\;a,b\;\in \;M\\ u_{p}^{2}\delta_{ab},\;\mathrm{Otherwise}. \end{array}$$

Similarly, the inference for $\sigma^{2}$ can be computed from Equation (24) as

(A31) $$\sigma^{2}=\frac{1}{N_{0}}\int Q\left(\underline{h}\right)\left(\|\underline{y}-\;\bar{A}\underline{h}{\|}^{2}\right)d\underline{h}$$

where

$${\hat{h}}_{g}=\{\begin{array}{c} \;h_{g}^{\mathrm{MAP}}\;\mathrm{if}\;g\;\in \;M\\ u_{g}\;\mathrm{if}\;g\;\in \;P \end{array}$$

The core procedure of the proposed CMF method is based on $L_{1}$-optimal sparsity parameters. The estimated sources are discovered by multiplying the respective rows of the $W^{k}\left(\omega \right)$ components with the corresponding columns of the $H^{k}\left(t\right)$ weights and time-varying phrase spectrum $e^{j\phi^{k}\left(\omega ,t\right)}$. The separated sources ${\hat{s}}_{j}\left(t\right)$ are obtained by converting the time-frequency represented sources into time domain.
